# 72399 Epigenetic Modification of Macrophages Contribute to Protective Memory in Against Staphylococcus aureus

**DOI:** 10.1017/cts.2021.448

**Published:** 2021-03-30

**Authors:** Mateo Pellegrini, Liana C. Chan, Colin Farrell, Scott G. Filler, Hong Lee, Vance G. Fowler, Elaine F. Reed, Michael R. Yeaman

**Affiliations:** 1 Lundquist Institute for Biomedical Innovation at Harbor-UCLA, University of California, Los Angeles, CA, David Geffen SOM; 2 University of California, Los Angeles, CA; 3 Lundquist Institute for Biomedical Innovation at Harbor-UCLA; 4 Duke University

## Abstract

ABSTRACT IMPACT: This work may provide new targets for vaccine and immunotherapeutic development against MRSA infections. OBJECTIVES/GOALS: Staphylococcus aureus is the leading cause of skin and skin structure infection (SSSI), a primary portal of entry for invasive infection. Patients with SA SSSI have a high 1-year recurrence. We have shown innate memory protects mice against SA SSSI. The goal of this project is to determine epigenetic mechanisms of protective memory against SA SSSI. METHODS/STUDY POPULATION: We have shown macrophages (Mf) afford protective memory against recurrent SA SSSI in mice. Priming by prior infection reduced skin lesion size and MRSA burden, which correlated with increased Mf in abscesses and lymph nodes. Priming potentiated the opsonophagocytic killing of SA by bone-marrow derived Mf (BMDM) in vitro, and their adoptive transfer into naive skin afforded protective efficacy in vivo. Here, we investigated epigenetic mechanisms of anti-SA efficacy in BMDMs. BMDM from naive (uninfected) or primed (SA SSSI) wild-type C57Bl/6 mice were cultured ex vivo. DNA from BMDM groups were isolated and analyzed for methylation changes using reduced representation bisulfite sequencing (RRBS). Pathway analyses of methylation changes were determined with Panther. RESULTS/ANTICIPATED RESULTS: Present findings indicate the protective memory afforded by BMDM was mediated by epigenetic modifications of the DNA. Using RRBS, we profiled differentially methylated regions (DMR) in DNA from naive vs. primed BMDM. Primed BMDM exhibited significantly different DMRs as compared to naive BMDM. Proximity to known genes were mapped using GREAT. Pathway analyses revealed DMRs predominant in genes integral to immune modulation, such as integrin signaling, cytokine/chemokine networks, and growth regulation. For example, SA-primed BMDM were hypermethylated proximate to GIMAP8 versus naive BMDM, suggesting repression of this protein. Gimap family ligands are small GTPase immune-associated proteins expressed in immune cells known to regulate macrophage lysosomal fusion during parasite infection. DISCUSSION/SIGNIFICANCE OF FINDINGS: These findings reveal epigenetic mechanisms of macrophage innate memory against recurrent MRSA infection. Functional testing of these genes in response to SA infection is needed to confirm their protective role. These insights may provide new targets for vaccine and immunotherapeutic development against MRSA.

